# *In Vivo* Tractography of Fetal Association Fibers

**DOI:** 10.1371/journal.pone.0119536

**Published:** 2015-03-05

**Authors:** Christian Mitter, Daniela Prayer, Peter C. Brugger, Michael Weber, Gregor Kasprian

**Affiliations:** 1 Department of Biomedical Imaging and Image-guided Therapy, Division of Neuroradiology and Musculoskeletal Radiology, Medical University of Vienna, Vienna, Austria; 2 Department of Systematic Anatomy, Center for Anatomy and Cell Biology, Medical University of Vienna, Vienna, Austria; University Medical Center Utrecht, NETHERLANDS

## Abstract

Association fibers connect different cortical areas within the same hemisphere and constitute an essential anatomical substrate for a diverse range of higher cognitive functions. So far a comprehensive description of the prenatal in vivo morphology of these functionally important pathways is lacking. In the present study, diffusion tensor imaging (DTI) and tractography were used to visualize major association fiber tracts and the fornix in utero in preselected non-motion degraded DTI datasets of 24 living unsedated fetuses between 20 and 34 gestational weeks (GW). The uncinate fasciculus and inferior fronto-occipital fasciculus were depicted as early as 20 GW, while in vivo 3D visualization of the inferior longitudinal fasciculus, cingulum and fornix was successful in older fetuses during the third trimester. Provided optimal scanning conditions, in utero DTI and tractography have the potential to provide a more accurate anatomical definition of developing neuronal networks in the human fetal brain. Knowledge about the normal prenatal 3D association tract morphology may serve as reference for their assessment in common developmental diseases.

## Introduction

Association fiber tracts connect cortical areas within the same hemisphere and are essential for the integrative function of distributed large-scale neurocognitive networks [1, 2]. Their damage can lead to disconnection syndromes resulting in diverse neuropsychological deficits, ranging from aphasic syndromes to disorders of attention or visual object recognition [3–8]. Because of this fundamental contribution of proper connectivity to brain function, diffusion tensor imaging (DTI) and tractography are potentially useful tools in the diagnosis and assessment of a wide range of developmental brain pathologies.

Tractography has been used to identify association fiber tracts in healthy adults [9–13], children and adolescents [14, 15], newborns [16] and preterm newborns [17]. Although white matter myelination begins during the fetal period in humans, it is a predominantly postnatal process, especially in association fibers [18, 19]. Initially, anisotropy in fiber tracts was thought to depend primarily on the development of myelin, but it was later found that early diffusion anisotropy in unmyelinated tissue substantially relates to the integrity of the axonal membrane [20], and can be influenced by other factors, including non-structural changes in voltage-gated sodium channel activity [21, 22] or oligodendrocyte maturation processes [23]. Due to these “premyelinating processes” [22], it is possible to investigate even unmyelinated developing fiber tracts in vivo in the preterm or fetal brain [17, 24–27].

In the human fetal brain tractography of association fiber tracts has already been demonstrated in post-mortem brain tissue [28–33]. However, after death the condition of the brain is almost inevitably altered due to microstructural changes, such as cell lysis, brain edema, or the fixation process itself [34], and macrostructural changes, such as the damage of fragile structures during autopsy and three-dimensional changes in morphology due to the loss of supporting structures like the skull and the meninges. These problems can be avoided by the examination of developing white matter fiber tracts in the living human fetus in utero using fetal MRI. Fetal MRI is a safe and well-established imaging method in prenatal diagnostics that is usually performed after 17 gestational weeks (GW) [35–37]. Using fetal MRI in utero tractography of major fiber tracts like the corpus callosum, thalamocortical and corticospinal tracts is possible as early as 18 GW [26]. The purpose of this study was to extend in utero tractography in the living human fetus to other fiber tracts that have been identified in the adult human brain and postmortem fetal DTI studies. These include association fiber tracts (uncinate fasciculus—UF, inferior fronto-occipital fasciculus—IFOF, inferior longitudinal fasciculus—ILF and the cingulum) as well as the fornix. The aims of this exploratory cross-sectional study were to demonstrate the three-dimensional morphology of these fiber tracts in the living human fetus in utero at early and late gestational stages, as well as to look for potential differences in diffusion parameters between different fiber tracts.

## Methods

### Subjects

The DTI datasets of 120 fetal MRI examinations were retrospectively inspected, and cases with inhomogeneous or low signal-to-noise ratios and/or gross fetal or maternal motion were excluded from further analysis. In 24 cases (20% of examinations) DTI data quality met these inclusion criteria. Fetal subjects were aged 20–34 gestational weeks (GW, calculated from the first day of the woman’s last menstrual cycle) at the time of fetal MRI examination. Post-menstrual fetal age was determined with reference to a previous sonography examination and was distributed as shown in Table 1. From one fetus 2 fetal MRI examinations, one at 22 and a second at 30 GW are included in this study. They are referred to as “subject 3” and “subject 17” respectively.

**Table 1:** Successful tractography results for individual subjects in left (L) and right (R) hemisphere.

Subject number	Gestational weeks (GW)	Uncinate fasciculus (UF)	Inferior fronto-occipital fasciculus (IFOF)	Inferior longitudinal fasciculus (ILF)	Cingulum	Fornix
1	20	L+R	L+R°			
2	21	L	L°			
3	22	L°	L			
4	23	L+R	L+R	L		
5	23	L+R				
6	24	R	L*+R*			
7	24	L+R	L			
8	24	L*+R*	L+R*			
9	24	L*+R	L*+R*			
10	25	L	L			
11	25	L				
12	26	L+R	L+R*			
13	26	R*	R*			
14	27	L+R				L+R
15	27	R°	R°		L+R	
16	28	L+R	L+R		L+R	
17	30	L°+R	L		L+R	L°+R*
18	30	L+R*°	R	L*		L
19	33	R	L+R	L+R	L+R	
20	33	L+R	L+R	R	L+R	L+R
21	33	L+R	L+R		L+R	L+R
22	33	L+R	L+R*°	L*	L+R	L+R
23	33	R	R*		L*+R*	L*+R*
24	34	L+R	L+R	R	L+R	L° R°

A fiber tract visualized with the standard FA = 0.15/angle = 27° parameters is depicted as an L or R. Use of an FA threshold of 0.10 is symbolized by a star (*). Use of an angle threshold of 45° is symbolized by a degree symbol (°).

Imaging was performed during routine clinical fetal MR examinations to exclude or confirm sonographically detected extracerebral fetal pathologies. Age appropriate morphology of supratentorial gray and white matter structures was confirmed by T2 and T1 weighted sequences. Diagnoses according to fetal MR included: renal agenesis (n = 2); multicystic kidneys (n = 2); scoliosis (n = 1); congenital diaphragmatic hernia (n = 2); hepatic cyst (n = 1); tetralogy of Fallot and small for gestational age cerebellum (n = 1); gastroschisis (n = 2); pulmonary hypoplasia (n = 1); cleft palate (= 1); blood ingestion (n = 1); cyst of the adrenal gland (n = 1); pathology of the liver and gastrointestinal tract (n = 1); plexus cyst without ventriculomegaly (n = 1) and normal fetal development (n = 6). All imaged mothers gave written, informed consent and the study was approved by the Ethics Committee of the Medical University of Vienna (EK Nr.:650/2010).

### Imaging protocol

All subjects underwent a fetal MRI examination for diagnostic purposes and were imaged on a 1.5 T(esla) Philips Achieva MR system (Philips Gyroscan, Best, The Netherlands) using a five-channel phased-array cardiac coil, adjusted to the position of the fetal head. In addition to the standard diagnostic protocol, an echo planar diffusion tensor sequence (repetition time TR = 2260msec, echo time TE = 90msec, b-values of 0 and 700sec/mm^2^), using 16 gradient-encoding directions, was acquired in an axial plane perpendicular to the axis of the brainstem. Fifteen slices were recorded during an overall imaging time of 1:16 minutes of scanning. The acquired voxel size of 2.14(axial)/2.19(sagittal)/3(slice thickness)mm was reconstructed to 0.94/0.94/3mm using an imaging matrix of 256. The specific absorption rate (SAR) did not exceed 11%/0.4W/kg, and thus, was within current safety recommendations [38, 39]. Generally, fetal DTI sequences in non-sedated fetuses were only acquired if fetal motion during acquisition of preceding structural sequences did not exceed certain (acceptable) limits. Standardized axial T2-w sequences (TR = 8828msec, TE = 140msec), with a voxel size of 0.75/0.75/3mm, or steady state free precession sequences (SSFP) (TR = 3.2msec, TE = 1.62msec) were acquired as anatomical references for tractography.

### Postprocessing and tractography

Using Philips Extended MR WorkSpace 2.6.3.3, a fractional anisotropy (FA) color-coded map was generated. Region of interest (ROI) placement was performed on diffusion weighted imaging (DWI) images using the diffusion weighted raw data as well as on geometrically coregistered T2-w or SSFP sequences. A deterministic multiple-ROI approach was used with a minimum of two ROIs placed within the projection path of the fiber tract of interest in accordance with previously published descriptions in the postmortem fetal tractography literature [28–33]. This was done for the following fiber tracts: uncinate fasciculus, inferior fronto-occipital fasciculus, inferior longitudinal fasciculus, cingulum and fornix. For tractography, a deterministic linear tracking algorithm [40] with the following cutoff values was deployed: min FA (FA threshold) 0.10–0.15; max angle change (angle threshold) 27.0–45.0 degrees; and min fiber length 10 mm. Tractography was first attempted with minimum thresholds of FA = 0.15 and angle = 27°. If tractography was not satisfactory with those standard parameters, the FA threshold was lowered to 0.10 and/or the angle threshold was increased to 45°.

After tractography whole-tract FA values were computed for the UF and IFOF using Philips Extended MR WorkSpace. In cases in which the IFOF could not be visualized as a single fiber tract, the individual FA values of IFOF components were converted into whole-tract FA values according to the following conversion algorithm: FA = (FA_1_ x Voxel_1_ + FA_2_ x Voxel_2_ +…+ FA_n_ x Voxel_n_)/(Voxel_1_ + Voxel_2_ +…+ Voxel_n_). With “n” representing the number of components that tractography of a fiber tract resulted in, “FA” the FA-value of the fiber tract component (1-n) and “voxel” the number of voxels that the fiber tract component (1-n) occupies.

### Statistical analysis

All statistical computations were performed using IBM SPSS Statistics (version 19.0). Mixed model ANOVA using full information maximum likelihood (FIML) was calculated to compare mean FA values between fiber tracts as well as left and right hemisphere for UF and IFOF. There was no data imputation. Full information maximum likelihood estimation under the missing at random (MAR) assumption [41] was used to deal with missing data. That is, the estimation routine uses all available data without discarding incomplete observations [42]. A p value of p< = 0.05 was considered to indicate significant results. Metric data like FA values are presented using means+/- SD. Nominal data are presented using absolute numbers and percentages.

## Results

### Feasibility of in utero tractography to visualize fetal association fiber tracts

Table 1 provides an overview over the feasibility of in utero tractography to visualize association fiber tracts in the 24 DTI examinations included in this study. Since the present study used a preselected cohort of non-motion degraded DTI examinations, the resulting numbers do not represent the true feasibility to visualize association fiber tracts in a random clinical fetal MR examination (80% of data discarded and excluded from further analysis).

**Fig 1:**
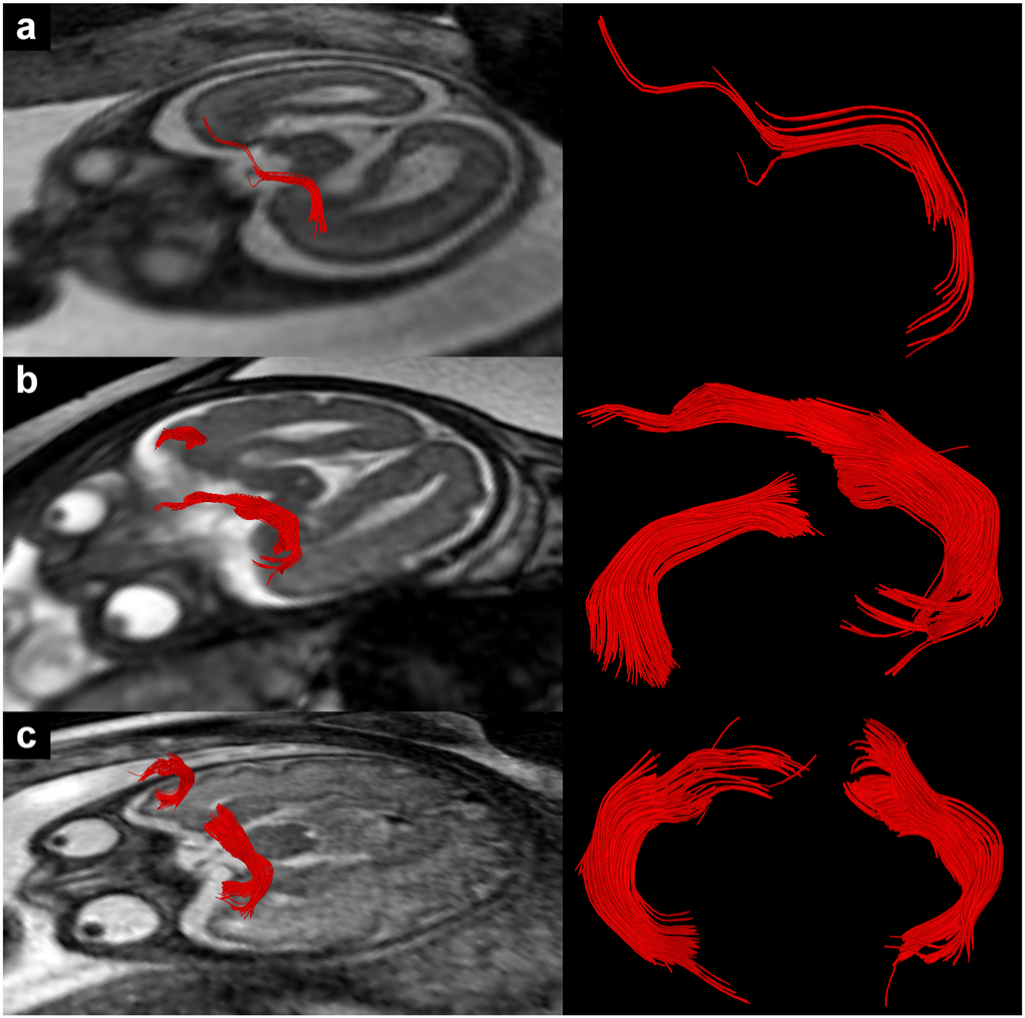
In utero tractography of the UF in subjects 3 (a), 16 (b) and 24 (c) (22, 28 and 34 GW respectively). Fiber tracts are projected on axial T2-weighted images.

**Fig 2:**
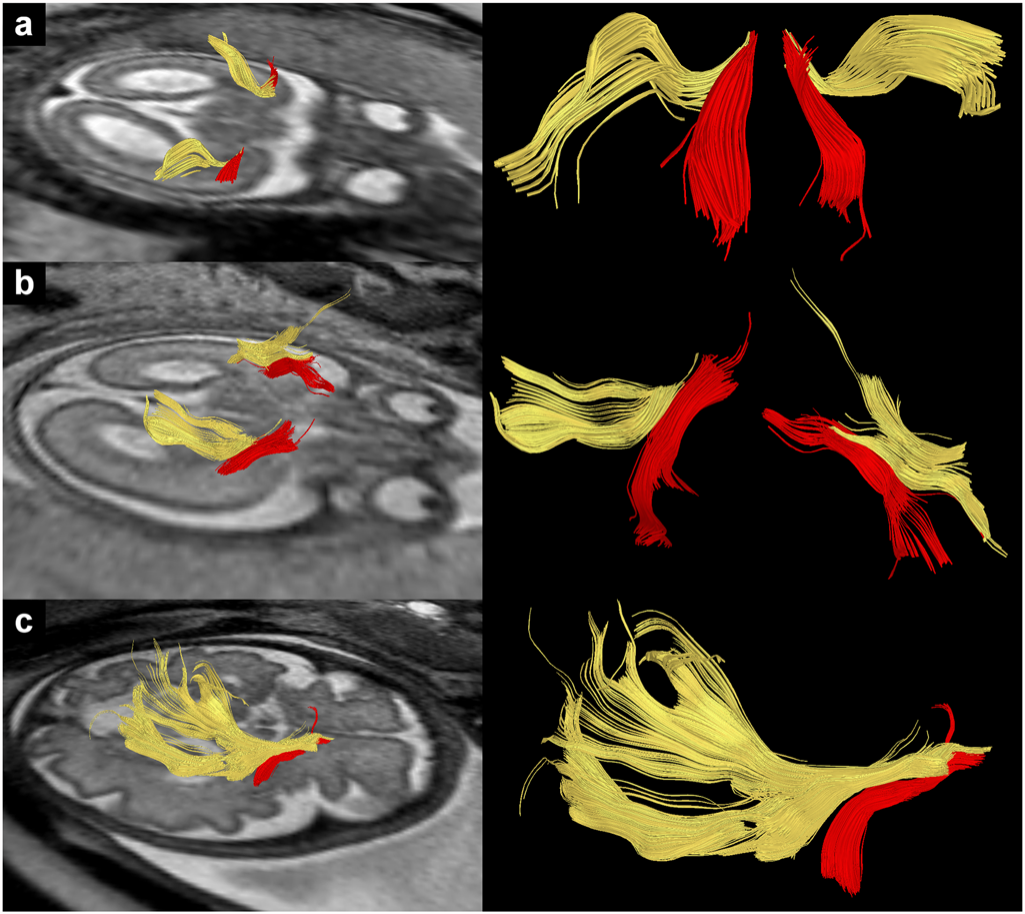
In utero tractography of the IFOF (yellow) in subjects 1 (a), 9 (b) and 23 (c) (20, 24 and 33 GW respectively) and its relation to the UF (red). Fiber tracts are projected on axial T2-weighted images.

For the UF and the IFOF there were no differences in the feasibility of tractography in relation to gestational age. Both fiber tracts could be successfully visualized as early as 20GW up until 34GW. Tractography of UF fiber tracts was possible in all 24 subjects (total 39 fiber tracts), in 19 subjects in the left hemisphere and in 20 subjects in the right hemisphere (Fig. 1). Visualization of IFOF fiber tracts was successful in 21/24 subjects (total 33 fiber tracts), in 17 in the left hemisphere and in 16 in the right hemisphere (Fig. 2). In 23/33 cases the IFOF could be visualized as a single fiber tract, whereas in the remaining 10/33 cases tractography resulted in a fiber tract consisting of more than one components. For statistical analysis, individual FA values of the components were converted into whole-tract FA values according to the conversion algorithm described in the methods section.

**Fig 3:**
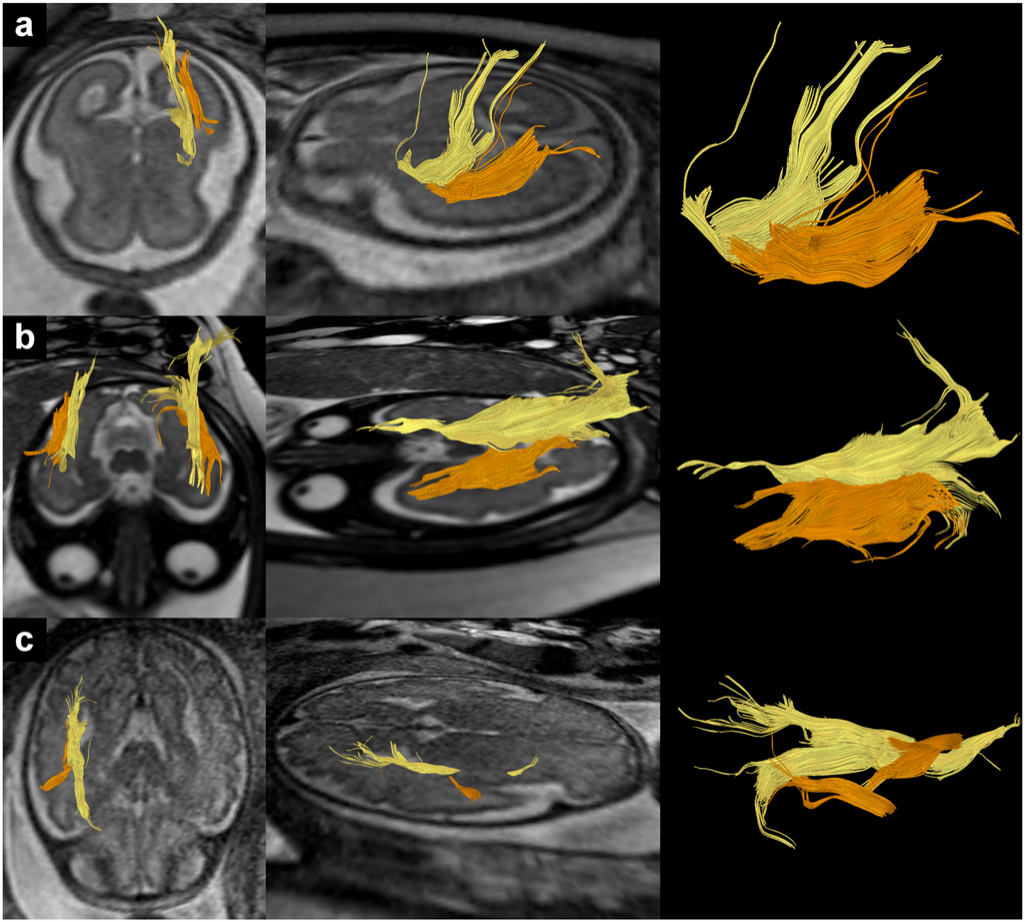
In utero tractography of the ILF (orange) in subjects 4 (a), 19 (b) and 24 (c) (23, 33 and 34 GW respectively) and its relation to the IFOF (yellow). Fiber tracts are projected on axial T2-weighted images.

In contrast, successful tractography of the other fiber tracts investigated in this study was strongly dependent on gestational age. Tractography of the ILF was only possible in 6/24 subjects (total 7 fiber tracts), in 4 in the left and in 3 in the right hemisphere (Fig. 3). Although the ILF could be found in 1 subject as early as 23GW (subject 4, left hemisphere; see Fig. 3a), all other ILF fiber tracts identified in this study (6/7) were from fetuses aged 30GW or older.

**Fig 4:**
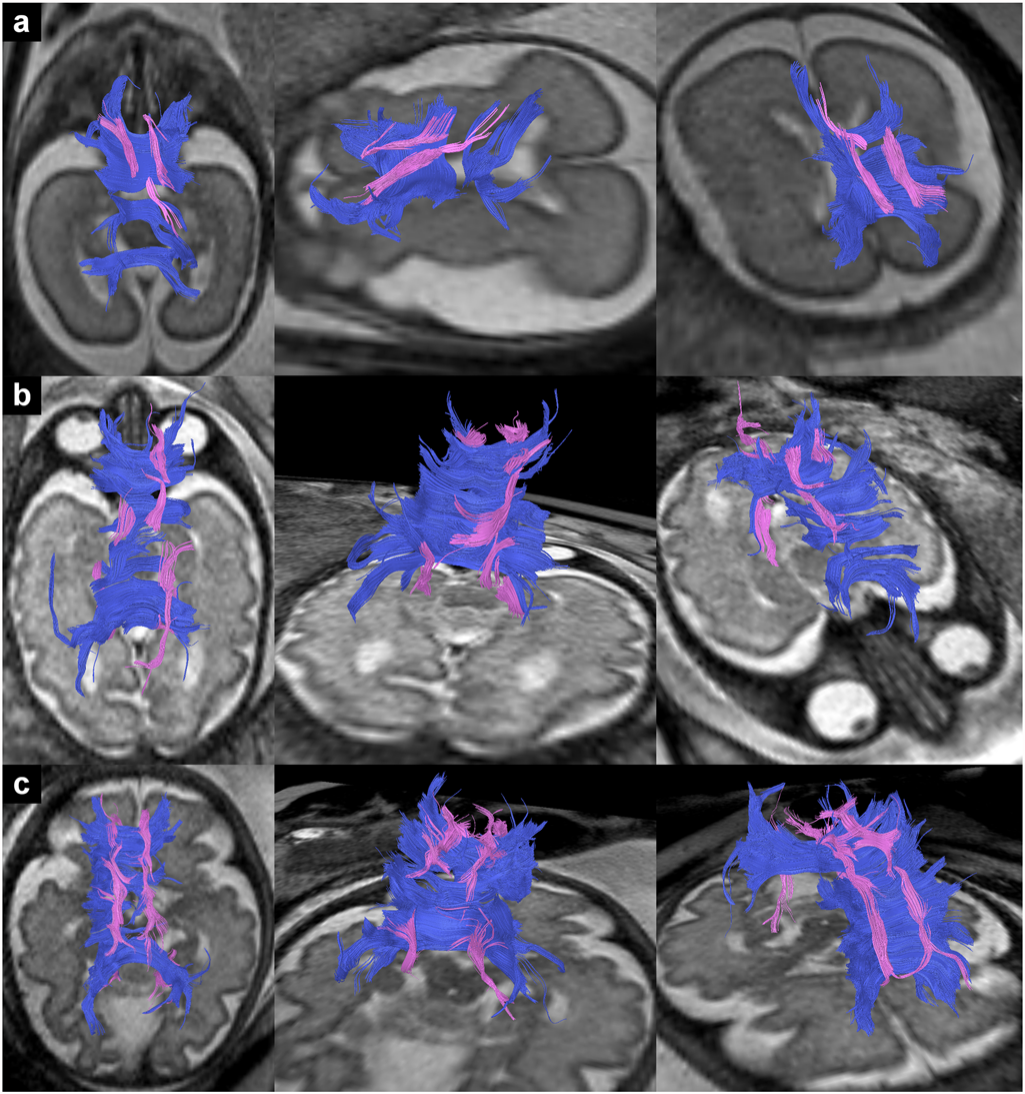
In utero tractography of the cingulum (pink) in subjects 15 (a), 20 (b) and 23 (c) (27, 33 and 33 GW respectively) and its relation to the corpus callosum (dark blue). Tractography of the corpus callosum was performed according to the methods described in Kasprian et al. (2008) [26]. Fiber tracts are projected on axial T2-weighted images.

Tractography of the cingulum and the fornix was only possible in older subjects with both fiber tracts identifiable from 27GW on. Tractography of the cingulum was successful in 9/24 subjects in both hemispheres (Fig. 4) with therefore a total of 18 fiber tracts. In 8/18 cases only 1 fiber tract component of the cingulum could be visualized whereas in 10/18 cases tractography resulted in more than one cingulum components.

**Fig 5:**
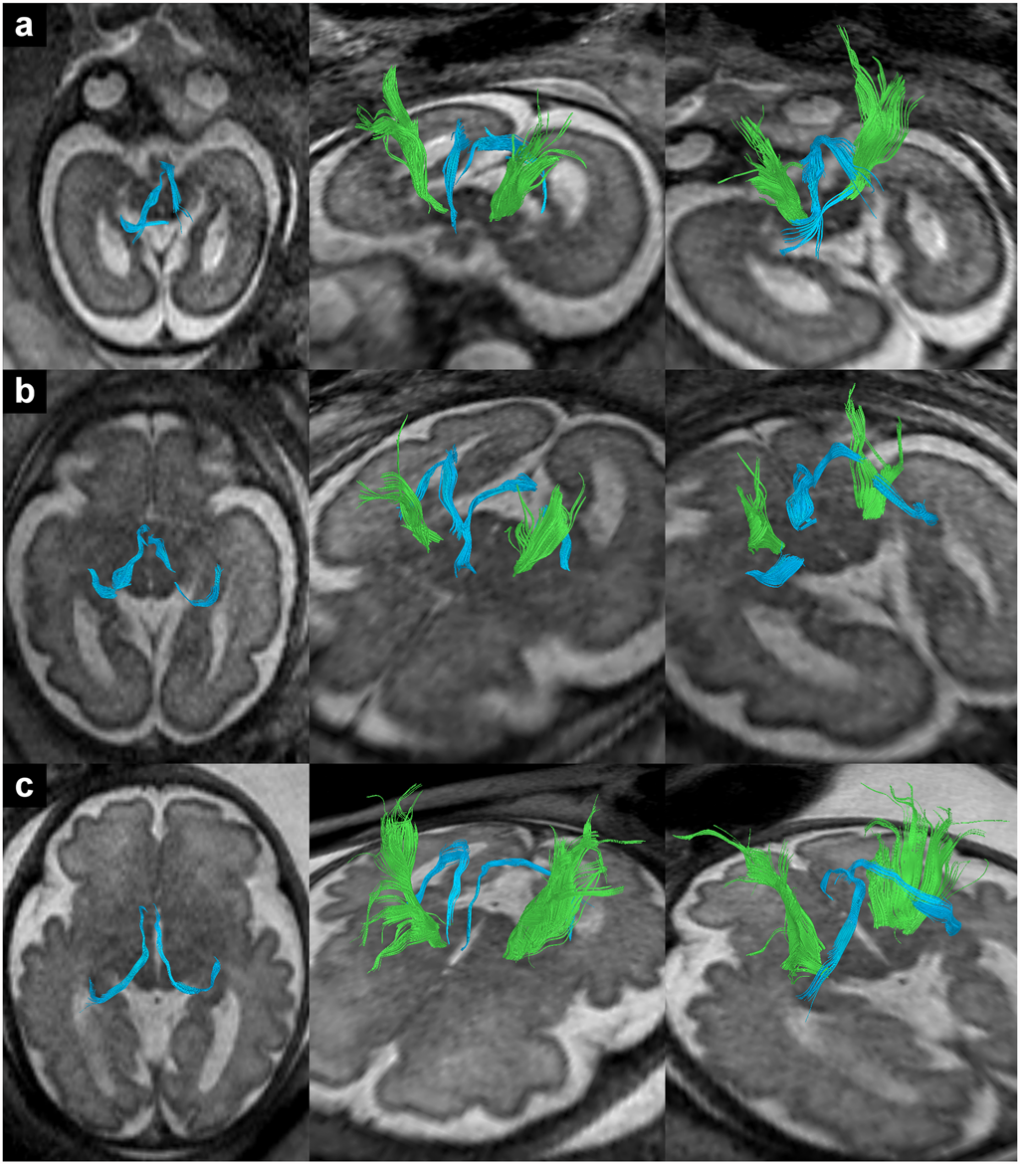
In utero tractography of the fornix (light blue) in subjects 14 (a), 17 (b) and 23 (c) (27, 30 and 33 GW respectively) and its relation to corticospinal/ corticopontine fibers (green). Tractography of corticospinal/ corticopontine fibers was performed according to the methods described in Kasprian et al. (2008) [26]. Fiber tracts are projected on axial T2-weighted images.

Visualization of the fornix was possible in 8/24 subjects (total 15 fiber tracts), in 7 subjects in both hemispheres and in 1 subject only in the left hemisphere (Fig. 5). In 5/15 cases the fornix was visualized as a single fiber tract and in 10/15 cases as a fiber tract consisting of more than one components.

The need for readjustment of FA and angle thresholds in certain cases was independent of gestational age (see Table 1).

### Quantitative analysis of differences in FA-value between fiber tracts

Because of the relative low number of subjects with successful tractography results for ILF, cingulum and fornix, the quantitative analysis of mean differences in FA-value between fiber tracts was restricted to UF and IFOF. Statistical analysis was performed using a mixed model ANOVA. Tests of model effects showed that mean FA differences between UF and IFOF fiber tracts could not be found in both hemispheres and mean FA differences between hemispheres could not be found for both fiber tracts (Table 2).

**Table 2:** Mean FA values for the left and right Uncinate fasciculus (UF) and Inferior fronto-occipital fasciculus (IFOF).

Mean FA	left	Right
UF	0,29	0,29
IFOF	0,30	0,27

There is a small but statistically significant difference between left and right IFOF as well as between UF and IFOF in the right hemisphere (see text).

### Mean differences in FA value between left and right UF and IFOF fiber tracts

Mean FA value (rounded) for the UF was 0.29 for the left and 0.29 for the right hemisphere, resulting in a mean difference of 0.00 (95% confidence interval: -0.02 to 0.02). This difference in mean FA-value was found to be statistically not significant (p = 0.966). On the other hand, for the IFOF a statistically significant (p<0.001) difference in mean FA-value of 0.03 between left and right hemisphere could be found (95% confidence interval: 0.01 to 0.04). With a mean of 0.30 the FA was found to be higher in the left hemisphere, compared to 0.27 in the right hemisphere.

### Mean differences in FA value between UF and IFOF of the same hemisphere

Between UF and IFOF of the right hemisphere a statistically significant (p = 0.036) difference in mean FA-value of 0.02 was found (95% confidence interval: 0.00 to 0.04) with the UF exhibiting a higher mean FA value than the IFOF. In the left hemisphere no statistically significant difference in mean FA between UF and IFOF was found (p = 0.261).

## Discussion

**Fig 6:**
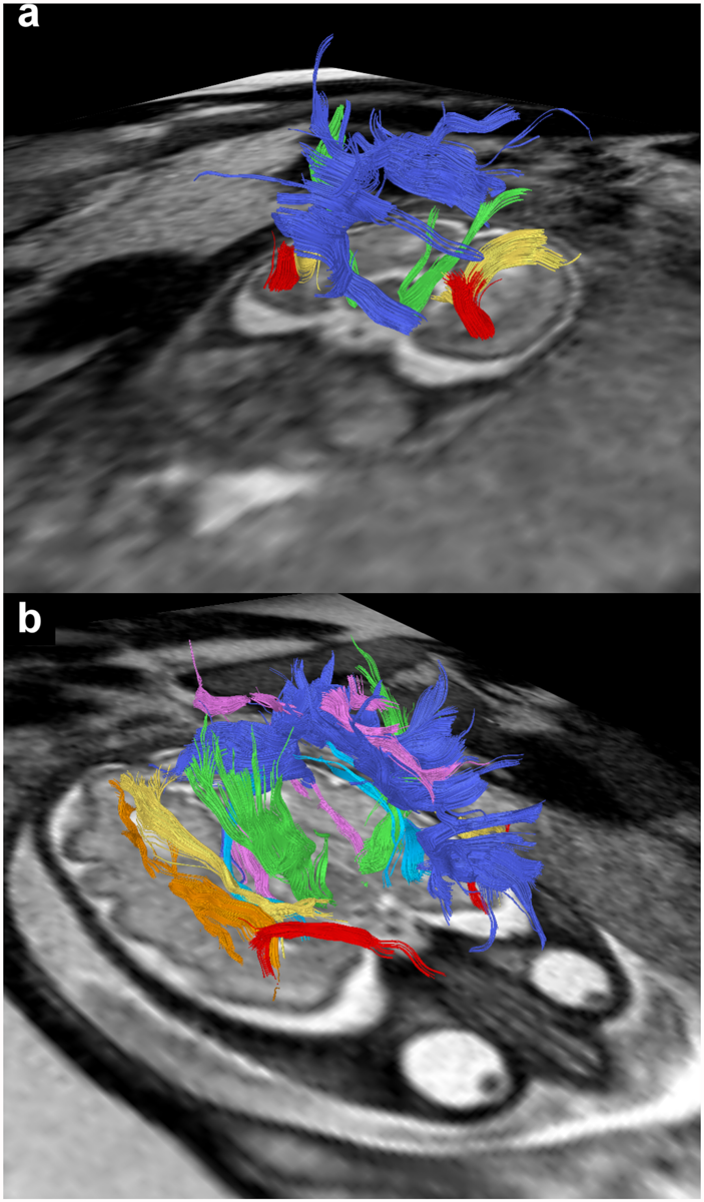
In utero tractography of association and limbic fiber tracts in subjects 1 (a) and 20 (b) (20 and 33 GW respectively) and their relation to the corpus callosum (dark blue) and corticospinal/ corticopontine fibers (green). Fiber tracts are projected on axial T2-weighted images. Visualized association fiber tracts include the UF (red), the IFOF (yellow), the ILF (orange) and the cingulum (pink). The limbic fiber bundle of the fornix is color-coded in light blue.

In utero DTI-based tractography can be used to visualize major association fiber tracts in living, unsedated fetuses as early as 20 GW. In contrast to post mortem DTI studies [28, 29, 31–33], which are biased by tissue degradation, particularly for developing subcortical structures [34], in utero DTI offers the possibility to investigate the developing fetal brain in its natural state and environment and allows the visualization of even subtle topographic relationships of distinct axonal pathways, that play important roles in the integrative function of distributed neural systems (Fig. 6).

### In utero tractography of the uncinate fasciculus (UF)

From all the association fiber tracts investigated in this study the UF turned out to be the most easily and reliably detectable one, with tractography being successful in all subjects at least in one hemisphere. The general appearance of the UF as visualized by in utero tractography corresponds well to published results of postmortem studies [29–32]. Both in utero and postmortem tractography are able to visualize the compact curved middle portion of the UF within the temporal stem and to a varying degree its continuation into the frontal and temporal lobes.

As can be seen in Fig. 1, fiber tracts in different subjects varied to some degree in length, cross-section and curvature. These differences in fiber tract appearance between subjects might be related to individual anatomical variations, but they might also be the result of differences in DTI data quality or thresholds for tractography. For example in subject 24 (c) (34 GW) tractography resulted in the visualization of a compact, c-shaped frontotemporal fiber tracts with a relative early subcortical breakup of streamlines, while in subject 16 (b) (28 GW) streamlines of the left UF continued much further into the frontal lobe and some appeared to enter the developing cortex. Since the same fiber propagation thresholds (FA = 0.15/angle = 27°) and tracking algorithm were used in both subjects, variations in the tract appearance cannot be attributed to differences in data postprocessing alone. Instead, the variability of tractography results most likely reflects current limitations of prenatal DTI data quality (low signal to noise, subtle movement, low resolution, non-isovoxel conditions). In addition, different tractography results between the left and right hemisphere could be related to physiological hemispheric asymmetries [43].

Continuing fiber development and the gradual dissolution of the subplate during the last third of gestation [44] may affect the possibility to trace fiber tract streamlines into the developing cortex. The relative low anisotropy observed within the subplate layer [45] of younger fetuses may represent an obstacle for conventional tractography methods and contribute to the subcortical breakup of fibers. More advanced DTI techniques, such as diffusion spectrum imaging [46, 47] may be able to address this limitation. However, due to the long acquisition times and high sensitivity to motion, these techniques are currently far from being applied as prenatal imaging techniques. In this study, no clear relationship between subcortical breakup of fibers and gestational age could be observed.

### In utero tractography of the inferior fronto-occipital fasciculus (IFOF)

In the present study the IFOF was the second most reliably detectable association fiber tract and was found immediately superior to the UF in the temporal stem with a similar fiber orientation to UF fibers. Posterior to the temporal stem region IFOF fibers separated from the UF and continued towards the sagittal stratum. Both the anteroposterior orientation of IFOF fibers and their location relative to the UF correspond to published results of postmortem fetal tractography [29, 31].

As can be seen from Fig. 2 the extension of the IFOF in the posterior direction varied substantially. Especially in younger fetuses IFOF streamlines often break up way before they reach the occipital lobe, sometimes shortly posterior to the temporal stem (see e.g. Fig. 2b). This subcortical breakup of IFOF fibers before they reach the occipital lobe corresponds to the postmortem findings of Huang et al. (2009) [29] in a 19 GW fetal brain, where IFOF fibers ended at about the level of the trigone of the lateral ventricle. It also corresponds to the results of postmortem IFOF tractography in the younger (19 and 22 GW) fetal brains investigated by Takahashi et al. (2012) [31]. Tractography in older subjects more often resulted in an IFOF that extended further posterior (see e.g. Fig. 2c). This is consistent with the findings of Takahashi et al. (2012) [31] that the IFOF as displayed by postmortem tractography becomes more prominent and longer with increasing gestational age, reaching its full length at 33 GW.

Within the posterior hemisphere visualized IFOF fibers were located closer to the ventricular surface than to the cortical plate. This would be consistent with a location of IFOF fibers within the fetal intermediate zone. Like the UF, and maybe related to the subplate layer’s low anisotropy [45], IFOF streamlines generally could not be followed to their projection areas within the cortical plate.

### In utero tractography of the inferior longitudinal fasciculus (ILF)

Contrary to the UF and the IFOF, in the present study successful in utero tractography of the ILF was rare and only possible in 25% of subjects. Tractography results for the ILF were located lateral and ventral to IFOF fibers in the middle/posterior temporal lobe (see Fig. 3). In the anterior temporal lobe ILF fibers continued to project towards the temporal pole, whereas IFOF fibers projected through the temporal stem into the ventral frontal lobe. The visual appearance of the ILF varied between subjects. In some cases (Fig. 3a and b) it presented as a long anteroposterior oriented fiber tract with a size similar to the IFOF, while in other cases (Fig. 3c) tractography was only able to visualize short fiber tract components within the projection path of the ILF.

Similar to the cingulum and the fornix, in utero tractography of the ILF was found to be strongly age-dependent, and in most cases only possible at 30 GW or older. This parallels the results of postmortem tractography in which visualization of the ILF was only successful in older brains [31]. However, the results of Huang et al. (2006) [29] suggest that tractography of small fiber clusters in the region of the ILF might also be possible in younger fetal brains. With 23 GW, subject 4 (Fig. 3a) represents the only fetus in this study in which visualization of the ILF was possible before 30 GW. The age dependency of ILF visualization may be related to fiber development, however, it is difficult to interpret this finding due to the relative low overall number of successful ILF tractographies in this study.

### In utero tractography of the cingulum

Successful in utero tractography of the cingulum was also strongly age-dependent and only possible in older fetuses between 27 and 34 GW. This contrasts with postmortem imaging studies in which tractography of the cingulum could be achieved as early as 19 GW [29, 31, 32]. The general appearance of in utero tractography of the cingulum corresponds to some degree to the results of postmortem studies, however there are also important differences. Contrary to post mortem tractography that visualizes the cingulum as a continuous, c-shaped fiber tract superior to the corpus callosum [29, 31, 32], in utero tractography depicted cingulum fibers as multiple short anteroposterior oriented fiber tracts with a breakup of streamlines in between them. Furthermore in some cases the cingulum could not be visualized in its entire length (e.g. see Fig. 4b, left hemisphere). The breakup of streamlines in between short fiber tract components is probably not related to the neuroanatomy of the fetal cingulum, but rather the result of the insensitivity of applied postprocessing algorithms to detect complex low anisotropic circular and curved pathways based on the present non-isovoxel thick-sliced DTI data. Therefore a discontinuous cingulum bundle should not be interpreted as the result of a pathological process. In some cases (see Fig. 4b and c) tractography of a fiber tract within the parahippocampal gyrus was possible, which may be the ventral part of the cingulum.

An interesting neuroanatomical feature of the cingulum is that it contains, apart from long association fibers, also short association fibers that enter and exit the fiber tract from various areas in the cingulate gyrus. This can be seen in histological tract-tracer studies [48, 49] as well as postmortem fetal tractography [29, 32]. The results of in utero tractography also mirror this finding. Along its entire course several groups of fibers seem to curve away from the cingulum and to project to areas within the cingulate gyrus.

### In utero tractography of the fornix

The general appearance of the fornix as visualized by in utero tractography corresponds to the results of postmortem fetal tractography studies [29–33]. Both in utero and postmortem tractography are able to visualize crus, corpus and columns of the fornix as a compact c-shaped fiber bundle. However, while postmortem tractography can perform this task as early as 13 GW [32], in utero imaging of the fornix in the present study was only successful in fetuses aged 27 GW or older.

Visualization of the fornix as a single compact fiber tract was not possible in all cases. Subject 17 (Fig. 5b) represents an example in which tractography resulted in a column/corpus and a crus component with a breakup of streamlines in between them, and in subject 14 (Fig. 5a) the crus of the fornix could not be visualized in the right hemisphere. The breakup of streamlines may be related to the relatively high curvature or the small diameter of this fiber tract. However, it could also be related to the hippocampal commissure, which is located between the crus and the corpus of the fornix [50]. Crossing commissural fibers could reduce the average anisotropy in this region and thereby interfere with tractography of the fornix as a single fiber tract. Interestingly in subject 14 (Fig. 5a) some fibers seem to diverge from the left fornix in this region and project towards the midline where they break up.

### Quantitative analysis of differences in FA-value between fiber tracts

One of the aims of the present study was to look for differences in diffusion parameters between different fiber tracts as well as between fiber tracts of the left and right hemisphere. No statistically significant difference in mean FA-value could be found between left and right UF with a mean FA value of about 0.29 for both hemispheres. Unfortunately there are no standard values for UF anisotropy in the fetal brain in vivo or postmortem. The mean FA values for the UF obtained in this study are similar to the mean FA values described for the sensorimotor tracts in the in utero tractography study of Kasprian et al. (2008) [26]. Dubois et al. (2006) [16] obtained DTI images of 1 to 4 month old healthy infants and found a mean FA value of 0.31 for the UF. In a sample of 6 to 17 year old children Eluvathingal et al. (2007) [14] measured mean FA values of 0.42. Anisotropy of the UF continues to increase during development and peaks at about 36 years [15].

For the IFOF the mean FA values measured in the present study were 0.30 in the left hemisphere and 0.27 in the right hemisphere. Again, these values are similar to mean FA values of the sensorimotor tracts in the fetal brain in utero [26]. Contrary to the UF, the difference in mean FA-value between left and right IFOF was found to be statistically significant. The higher anisotropy in the left hemisphere corresponds to the results of Eluvathingal et al. (2007) [14] in a sample of 6 to 17 year old children, where a trend towards higher FA in the left IFOF was detected. However, hemispheric differences in FA value of the IFOF were not detected in a study of infants who were born preterm [51].

FA differences between UF and IFOF may be related to differences in fiber tract microstructure. A denser packing of axons within the UF may result in a higher FA compared to the IFOF, whose fibers fan out in the sagittal plane when projecting toward the occipital lobe.

### Limitations and advantages of in utero fetal DTI

Previous DTI studies of developing association fiber tracts in the human fetus have relied on postmortem fetal brains [28, 29, 31–33] and used high magnetic field strength and long image acquisition times to generate high-resolution datasets. In contrast, because of fetal and maternal movements, MR examinations of living fetuses in utero rely on ultrafast sequences [52] and are typically performed on standard clinical 1.5T MRI scanners [53], which results in a lower spatial resolution compared to postmortem imaging. As the major issue in prenatal DTI is fetal motion [54], the skilled timing of image acquisition between periods of fetal movements and short acquisition times (below 2 minutes) are essential and result in acceptable DTI data quality for further postprocessing in a certain proportion of cases [26]. Improvements in motion correction [55] and sequence development might help to address this problem in the future and allow tractography in more cases than currently possible. Another limitation of DTI-based tractography is its inability to resolve crossing fibers. Although specialized techniques, such as HARDI [56] and q-ball imaging [57] could solve this problem, these techniques are not suitable for in utero imaging due to their long image acquisition times.

To date there are no standardized FA and angle threshold recommendations for tractography in the fetal brain. By lowering FA-thresholds and increasing angle-thresholds fiber tracts can be tracked more permissively, which increases the chance of successful tractography but also elevates the risk of visualizing false-positive pathways. Increasing FA and decreasing angle-thresholds leads to a more conservative tractography result, with a higher risk of unsuccessful tractography results and false-negatives.

Despite these limitations, in utero fetal DTI avoids the problem of postmortem tissue degradation and MR signal changes and offers the exciting possibility to investigate an intact developing brain in its natural environment. In utero tractography provides a three-dimensional visualization of developing fetal white matter that is not affected by changes in morphology due to the loss of supporting structures like the skull and the meninges after autopsy. As postnatal neonatal MRI is logistically demanding and frequently requires the use of sedation, prenatal imaging provides a reasonable and even less invasive alternative in the longitudinal assessment of early human brain development. The now proven potential of in utero DTI in the visualization of normal and abnormal fetal brain connectivity [58] supports further integration of fetal MRI in the perinatal diagnostic workup of fetuses with suspected brain pathologies.

## Conclusions

Provided optimal imaging conditions DTI-based tractography can be used to visualize the morphological appearance of major association fiber tracts in the developing fetal brain in utero. Identifiable fiber tracts include the UF and the IFOF as early as 20 GW, and the ILF, the cingulum and the fornix in older fetuses. The results of in utero tractography generally correspond to already available post mortem fetal imaging data. Quantitative analysis of diffusion parameters provides preliminary evidence for asymmetric development of certain association fiber tracts.

The possibility to non-invasively investigate association fiber tracts in utero with DTI-based tractography may be useful for a more precise evaluation of intrauterine white matter damage and for the prognosis of postnatal neurological sequelae. However, it is evident that current technology and methods have their limitations, which have to be taken into account if in utero tractography is applied in a clinical setting.

## Supporting Information

S1 Fig Comparison of left and right Uncinate fasciculus (UF) FA-values for each subject plotted against gestational age. Mean FA values (rounded) for the UF were 0.29 for the left and 0.29 for the right hemisphere, resulting in a mean difference of 0.00 (95% confidence interval: -0.02 to 0.02). This difference in mean FA-value was found to be statistically not significant (p = 0.966).
(TIF)

S2 Fig Comparison of left and right Inferior fronto-occipital fasciculus (IFOF) FA-values for each subject plotted against gestational age. Mean FA values (rounded) for the IFOF were 0.30 for the left and 0.27 for the right hemisphere, resulting in a mean difference of 0.03 (95% confidence interval: 0.01 to 0.04). This difference in mean FA-value was found to be statistically significant (p<0.001).
(TIF)

S3 Fig Comparison of FA-values for UF and IFOF within the left hemisphere for each subject plotted against gestational age. In the left hemisphere no statistically significant difference in mean FA between UF and IFOF was found (p = 0.261).
(TIF)

S4 Fig Comparison of FA-values for UF and IFOF within the right hemisphere for each subject plotted against gestational age. Between UF and IFOF of the right hemisphere a statistically significant (p = 0.036) difference in mean FA-value of 0.02 was found (95% confidence interval: 0.00 to 0.04) with the UF exhibiting a higher mean FA value (0.29) than the IFOF (0.27).
(TIF)
