# Supplementary figures and images for: In Vivo Tractography of Fetal Association Fibers
Source: PLoS One. 2015 Mar 5;10(3):e0119536. doi: 10.1371/journal.pone.0119536 (PMC4350986; doi:10.1371/journal.pone.0119536)

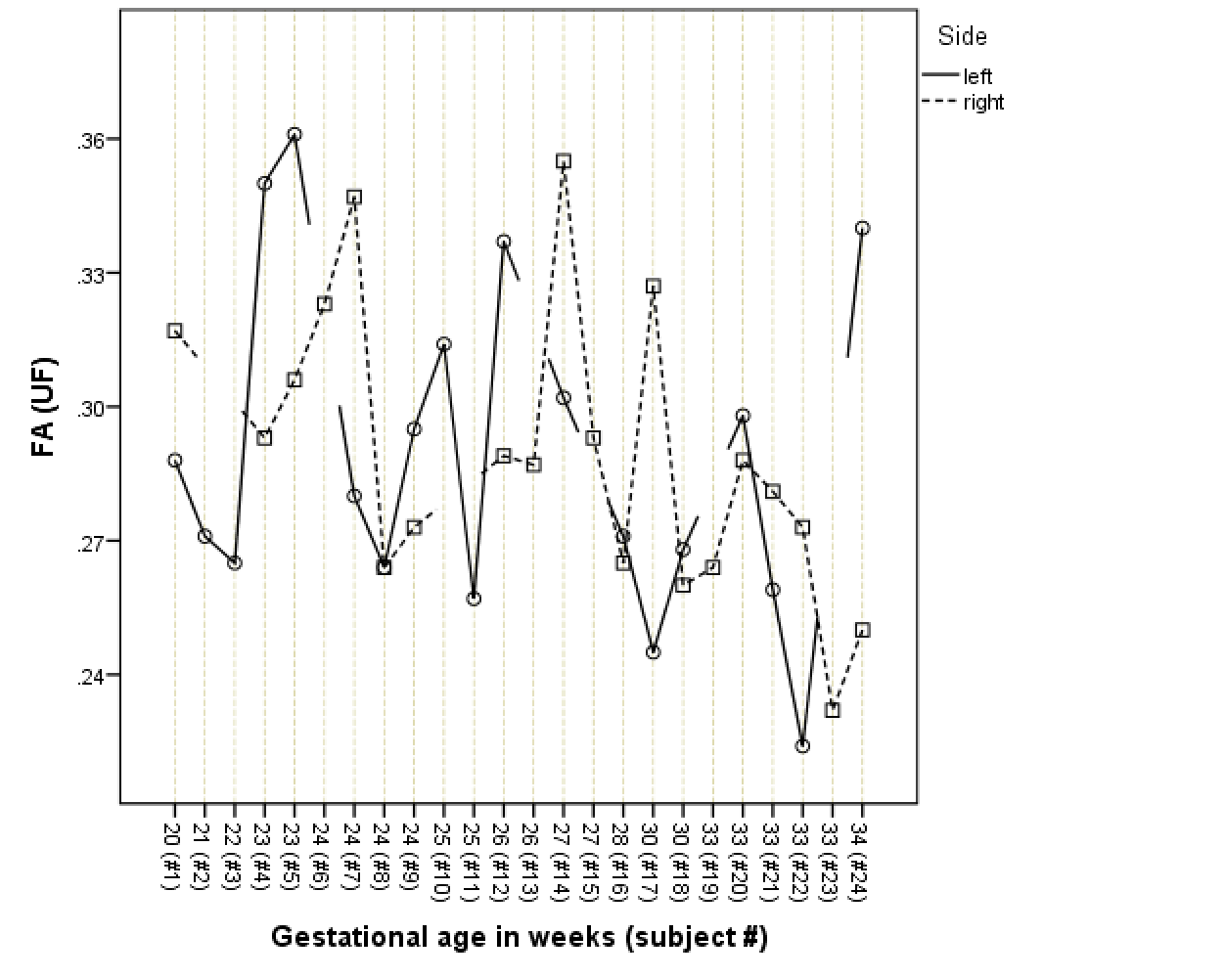

Supplement: S1 Fig — Mean FA values (rounded) for the UF were 0.29 for the left and 0.29 for the right hemisphere, resulting in a mean difference of 0.00 (95% confidence interval: -0.02 to 0.02). This difference in mean FA-value was found to be statistically not significant (p = 0.966). (TIF) [file pone.0119536.s001.tif]

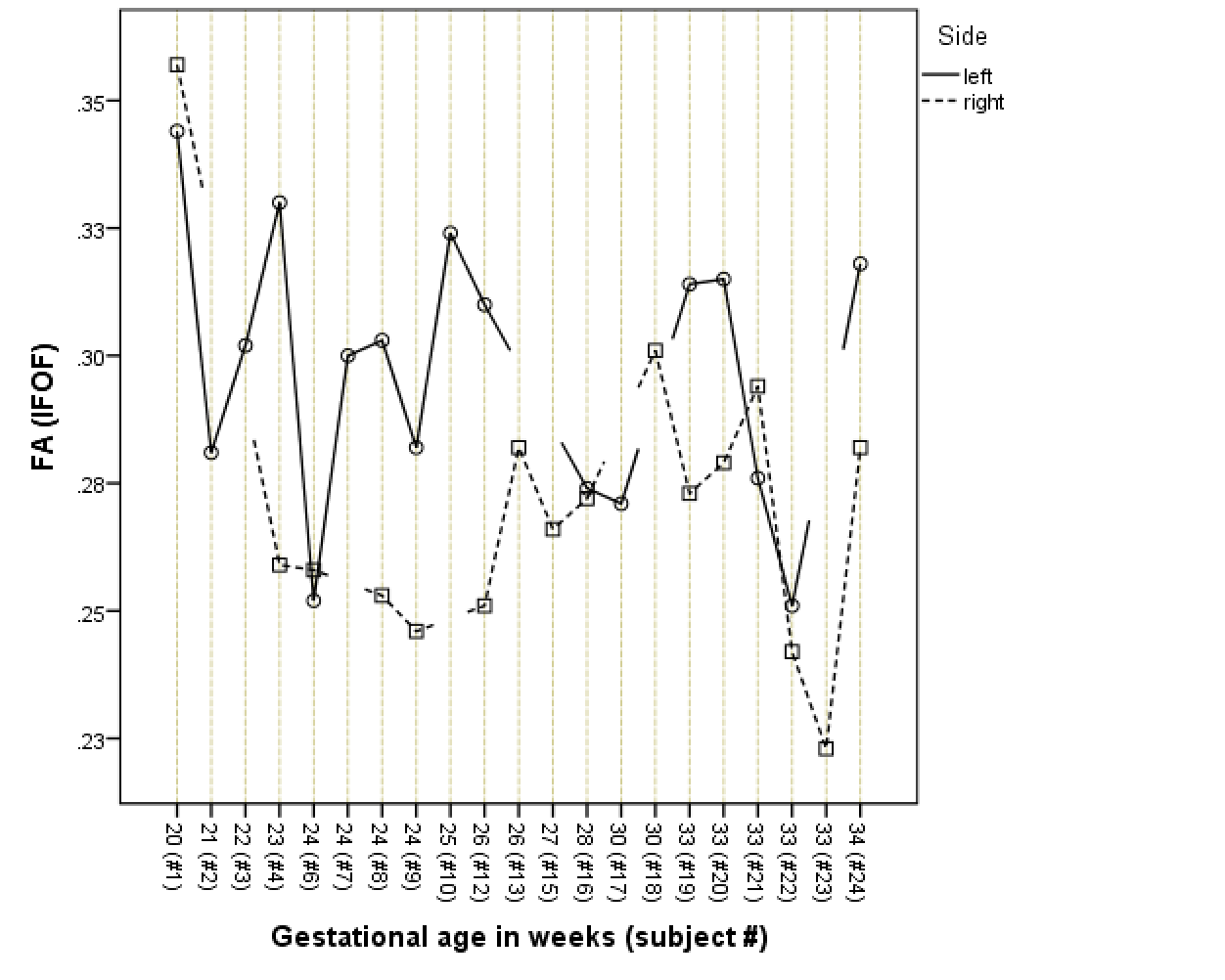

Supplement: S2 Fig — Mean FA values (rounded) for the IFOF were 0.30 for the left and 0.27 for the right hemisphere, resulting in a mean difference of 0.03 (95% confidence interval: 0.01 to 0.04). This difference in mean FA-value was found to be statistically significant (p<0.001). (TIF) [file pone.0119536.s002.tif]

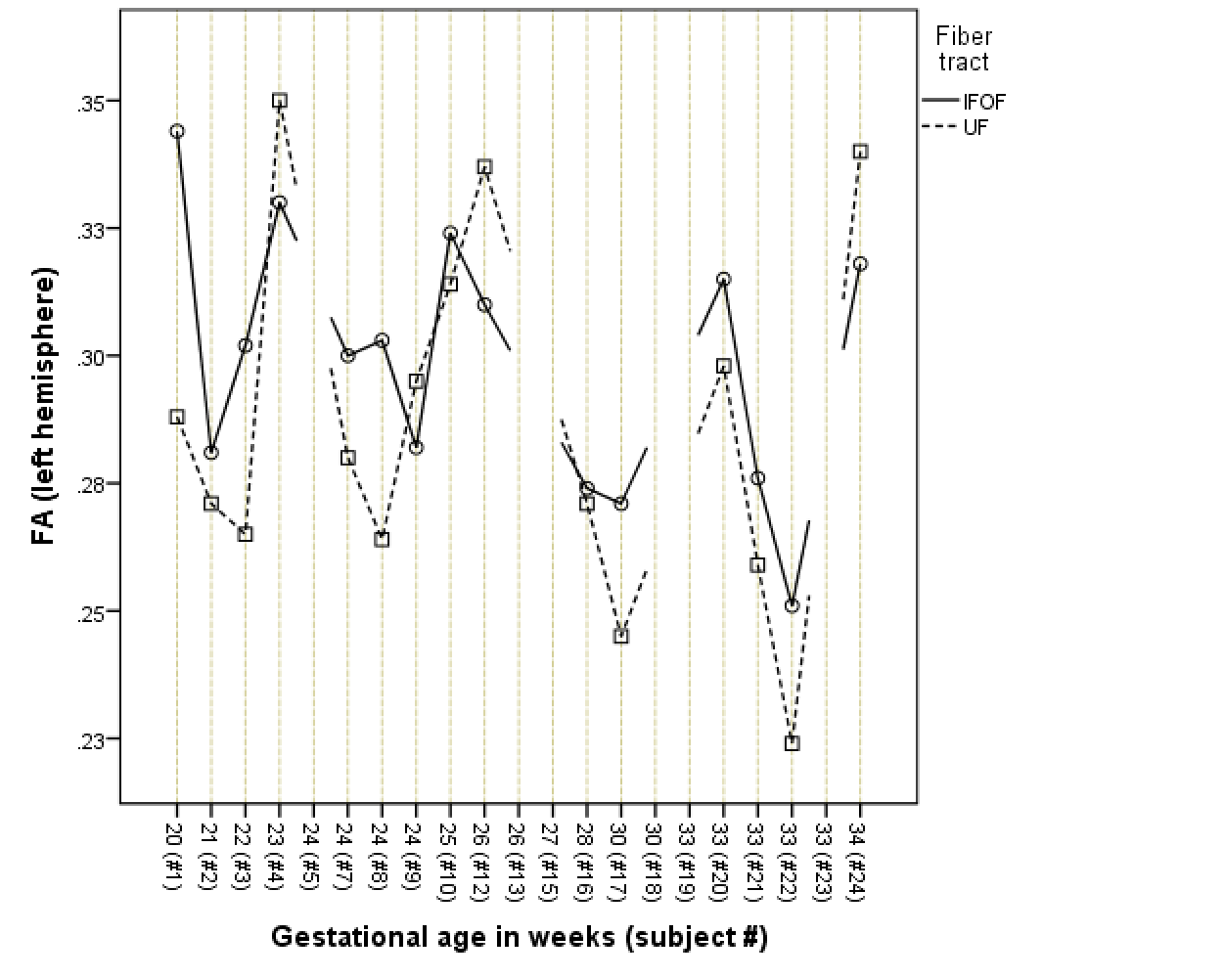

Supplement: S3 Fig — In the left hemisphere no statistically significant difference in mean FA between UF and IFOF was found (p = 0.261). (TIF) [file pone.0119536.s003.tif]

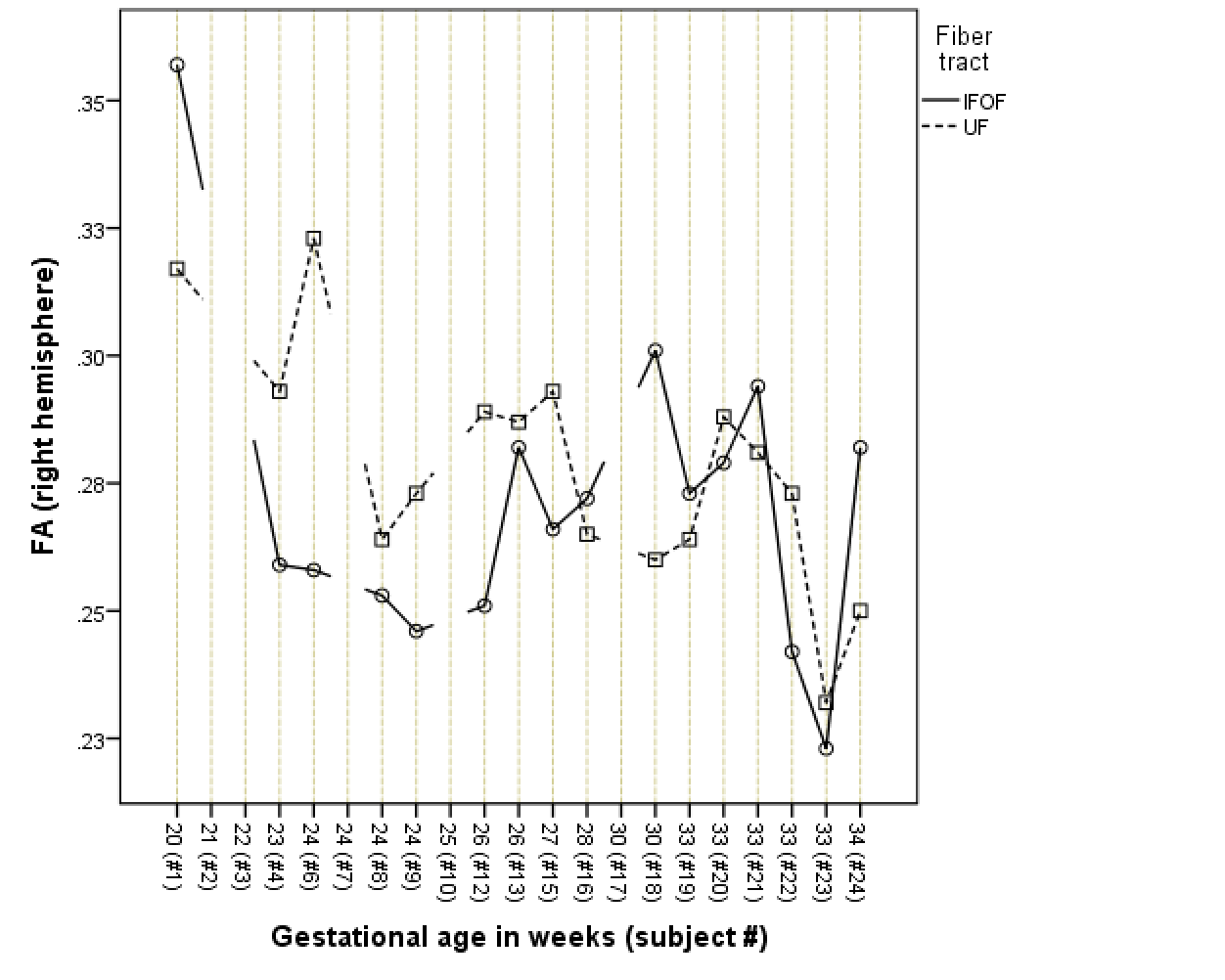

Supplement: S4 Fig — Between UF and IFOF of the right hemisphere a statistically significant (p = 0.036) difference in mean FA-value of 0.02 was found (95% confidence interval: 0.00 to 0.04) with the UF exhibiting a higher mean FA value (0.29) than the IFOF (0.27). (TIF) [file pone.0119536.s004.tif]
